# Gender differences in attitudes impeding colorectal cancer screening

**DOI:** 10.1186/1471-2458-13-500

**Published:** 2013-05-24

**Authors:** Paul Ritvo, Ronald E Myers, Lawrence Paszat, Mardie Serenity, Daniel F Perez, Linda Rabeneck

**Affiliations:** 1 School of Kinesiology and Health Science, York University, 136 Chemistry Building, 4700 Keele Street, Toronto, ON M3J 1P3, Canada; 2 Research, Prevention and Cancer Control, Cancer Care Ontario, 505 University Avenue, Toronto, ON M5G 2L7, Canada; 3 Division of Population Science, Department of Medical Oncology, Thomas Jefferson University, 1020 Walnut Street, Philadelphia, PA 19107, USA; 4 Institute for Clinical Evaluative Sciences, 2075 Bayview Avenue, Toronto, ON M4N 3M5, Canada; 5 Institute for Health Policy Management and Evaluation, Dalla Lana School of Public Health, University of Toronto, 155 College Street, Toronto, ON M5T 3M7, Canada; 6 Sunnybrook Health Sciences Centre, 2075 Bayview Avenue, Toronto, ON M4N 3M5, Canada; 7 Prevention and Cancer Control, 620 University Avenue, Toronto, ON M5G 2L7, Canada; 8 Department of Medicine, University of Toronto, 1 Kings College Circle, Toronto, ON M5S 1A8, Canada

## Abstract

**Background:**

Colorectal cancer screening (CRCS) is the only type of cancer screening where both genders reduce risks by similar proportions with identical procedures. It is an important context for examining gender differences in disease-prevention, as CRCS significantly reduces mortality via early detection and prevention. In efforts to increase screening adherence, there is increasing acknowledgment that obstructive attitudes prevent CRCS uptake. Precise identification of the gender differences in obstructive attitudes is necessary to improve uptake promotion. This study randomly sampled unscreened, screening - eligible individuals in Ontario, employing semi-structured interviews to elicit key differences in attitudinal obstructions towards colorectal cancer screening with the aim of deriving informative differences useful in planning promotions of screening uptake.

**Methods:**

N = 81 participants (49 females, 32 males), 50 years and above, with no prior CRCS, were contacted via random-digit telephone dialing, and consented via phone-mail contact. Altogether, N = 4,459 calls were made to yield N = 85 participants (1.9% response rate) of which N = 4 participants did not complete interviews. All subjects were eligible for free-of-charge CRCS in Ontario, and each was classified, via standard interview by CRCS screening decision-stage. Telephone-based, semi-structured interviews (SSIs) were employed to investigate gender differences in CRCS attitudes, using questions focused on 5 attitudinal domains: 1) Screening experience at the time of interview; 2) Barriers to adherence; 3) Predictors of Adherence; 4) Pain-anxiety experiences related to CRCS; 5) Gender-specific experiences re: CRCS, addressing all three modalities accessible through Ontario’s program: a) fecal occult blood testing; b) flexible sigmoidoscopy; c) colonoscopy.

**Results:**

Interview transcript analyses indicated divergent themes related to CRCS for each gender: 1) bodily intrusion, 2) perforation anxiety, and 3) embarrassment for females and; 1) avoidant procrastination with underlying fatalism, 2) unnecessary health care and 3) uncomfortable vulnerability for males. Respondents adopted similar attitudes towards fecal occult blood testing, flexible sigmoidoscopy and colonoscopy, and were comparable in decision stage across tests. Gender differences were neither closely tied to screening stage nor modality. Women had more consistent physician relationships, were more screening-knowledgeable and better able to articulate views on screening. Men reported less consistent physician relationships, were less knowledgeable and kept decision-making processes vague and emotionally distanced (i.e. at ‘arm’s length’).

**Conclusions:**

Marked differences were observed in obstructive CRCS attitudes per gender. Females articulated reservations about CRCS-associated distress and males suppressed negative views while ambiguously procrastinating about the task of completing screening. Future interventions could seek to reduce CRCS-related stress (females) and address the need to overcome procrastination (males).

## Background

Colorectal cancer screening (CRCS) is the only type of cancer screening where both genders receive identical procedures and confront comparable risks reduced by similar proportions. CRCS is an important context for examining male–female differences in disease-preventive behaviour, because it significantly reduces mortality via early detection (greater likelihood of effective, curative treatment) and prevention (colonoscopy-detected polyps are removable before they become malignant tumours) [1]. Because increased CRCS uptake rates are statistically proportional to reduced mortality [2-7], international efforts are aimed at increasing CRCS rates (e.g. in Canada, United States, Australia, United Kingdom, France, Israel, India, and Italy). Amidst the continual search for approaches that increase uptake, there is acknowledgment that obstructive attitudes impede CRCS [8-13]. Accordingly, precisely identifying screening-obstructive attitudes is necessary for devising interventions to overcome them.

Existing data suggest the obstructive attitudes that stop or delay CRCS and other preventive health behaviours differ by gender [9,14-21]. Accordingly, identification of gender-specific obstructions can be used to more precisely target and resolve the cognitions that impede use of life-improving, life-saving procedures. In addressing how males and females approach CRCS differently, the basic findings of poorer male health (than females), reflected in higher mortality rates [22,23] and the identification of male gender as a significant mortality risk are linked to the less frequent use of health services [24-27]. Even when males progress to health care contacts, they unfortunately spend less time during doctor visits than women, receiving less advice about risk factor reduction [24,27-30]. Male–female CRCS comparisons can help shed light on these deficits in male health care help-seeking given the greater acceptance of and use of health care services by women, and higher screening levels across female socioeconomic-ethnic groups [31,32]. The factors described above likely contributed to a recent finding in a population-based study of CRCS in 1013 adults (aged 50 years and above) where a larger proportion of males (55%) than females (46%) had never heard of Fecal Occult Blood Testing (FOBT), the typical entry-level CRCS modality [33,34].

Female-specific CRCS research, on the other hand, indicates aversion (when compared to men) to both endoscopic screening (internal scoping in flexible sigmoidoscopy or colonoscopy) and FOBT (fecal sampling) [35-45]. In the same population-based study cited above, of the individuals reporting FOBT awareness, 26% of females vs. 17% of males were not considering obtaining FOBT screening [33]. This pattern of greater female FOBT rejection was also found in a patient sample of primary care practice attendees in the US, by Myers et al. [46]. Other findings, however, have identified advantages for males. For example, more men than women attended screening (73% versus 67%) in a large UK community trial of Flexible Sigmoidoscopy (N = 5462) although the higher male attendance was partially explained by the lower levels of socioeconomic deprivation, higher levels of marital status and lower perceived screening barriers in male study participants [16]. Male gender was found to be a significant positive factor for screening uptake (CRC) in accordance with guideline recommendations in an Australian study within a population-based sample of First Degree Relatives (FDRs) of CRC-diagnosed individuals [47]. These male-advantage findings, however, are countered by results from a review of intervention studies meeting moderate methodological rigour where two trials with gender-based analyses demonstrated better effects in females than males [48-50], while in five other trials, no gender-based differences were found [51-55]. Another review focused specifically on participation equity in colorectal cancer screening amongst population subgroups: eight studies were identified that explored gender inequity in access/utilization of CRC screening, and in six of these a higher rate of screening participation was found amongst women compared to men, although screening test type, participant type (membership in high versus low risk groups), and age were found to interact with gender [56]. Two studies demonstrated partial results in that women were more likely to undertake FOBT but less likely to undertake invasive endoscopy or colonoscopy than males [57,58]. In still another review of studies [59] aimed at ascertaining CRC screening rates and impacting factors, 10 randomized trials [60-69] and seven non-randomized studies [70-76] were identified with FOBT screening outcomes while 8 studies were identified with sigmoidoscopy [69,71,76-81] and 2 with colonoscopy outcomes [79,82]; no systematic male–female differences emerged from these studies [59]. Altogether, while there seems a female advantage to some degree, the varying results suggest a need to better understand attitudinal obstacles for both sexes in the detailed descriptions that can be derived from qualitative findings.

Because better understandings of male–female reactions to CRCS can be useful in increasing uptake and informing other health promotion efforts, we undertook a qualitative, interview-based study to pinpoint gender-specific obstructive attitudes. Having directly completed survey studies on gender-related CRCS differences [33,34], we opted for qualitative study to more thoroughly investigate differences via semi-structured interviews, using a population-based recruitment. The transcribed interviews were analyzed to focally identify attitudinal obstructions related to three standard screening modalities (FOBT, Flexible Sigmoidoscopy, Colonoscopy) with the intent of deriving data useful in specifically tailoring CRCS promotions to each gender. As this study was undertaken in Canada, it is important to note that the Canadian Association of Gastroenterology recommended in a 2010 update [83] of their 2004 screening recommendations [84] that individuals of average risk participate in one or both of the following screening regimes: 1) FOBT every 1–2 years or 2) Flexible Sigmoidoscopy every 10 years or longer, with colonoscopy used when judged necessary. Recommendations for screening vary by region and are continuously reviewed, but generally follow a similar approach to Cancer Care Ontario’s *Colon Cancer Check,* (used here as an example) that individuals 50–75 years of age perform an FOBT every 1–2 years or receive a colonoscopy approximately every five to ten years depending on baseline risk.

## Methods

Telephone-based, semi-structured interviews (SSIs) were undertaken by trained interviewers to explore gender differences in attitudes towards CRCS. The SSI questions addressed three screening modalities accessible free-of-charge through Ontario’s screening program: a) FOBT; b) Flexible sigmoidoscopy; and c) Colonoscopy, and focused on 5 attitudinal domains selected to reflect attitudes that would obstruct or facilitate screening uptake, accenting frequently-cited obstructive perceptions (e.g. pain and anxiety) and conscious gender differences (and those the subject was not consciously aware of).

1) Screening Experience at the Time of Interview

2) Key Barriers to CRC Screening Adherence

3) Key Factors Predicting CRC Screening Adherence

4) Experience of Pain and Anxiety Reponses related to CRC Screening

5) Experience of Gender – Differences in CRC Screening

Standard descriptions and explanations of screening modalities were provided when participants indicated knowledge deficits, following the goal of helping participants respond from informed perspectives (based on their personal view of necessary information). This supplementation of current knowledge was carefully undertaken to avoid communicating biases favouring specific attitudes. (See Table 1 for Semi-structured interview schedule).

**Table 1:** Semi-structured interview schedule


**Patient’s current experience of CRC screening - FOBT**	
What are your current feelings about FOBT screening for colon cancer?	What do you know about FOBT screening and what do you feel are the most important points to know about?
What do you feel are the Pros and Cons of FOBT screening?	What do you feel are the important male – female differences in FOBT screening?
What do you see as the advantages of getting FOBT screening over not getting FOBT screening?	How do you feel you approach FOBT screening differently than a (male, female – whichever is the opposite sex)?
What do you see as the advantages of not getting FOBT screening over getting FOBT screening?	What do you feel are important points about FOBT screening that we may not have yet covered?
**Patient’s current experience of CRC screening - flexible sigmoidoscopy**	
What are your current feelings about Flexible Sigmoidoscopy (FS) screening for colon cancer?	What do you know about FS screening and what do you feel are the most important points to know about?
What do you feel are the Pros and Cons of FS screening?	What do you feel are the important male – female differences in FS screening?
What do you see as the advantages of getting screened with FS screening over not getting screened with FS screening?	How do you feel you approach FS screening differently than a (male, female – whichever is identified as the opposite sex)?
What do you see as the advantages of not getting screened with FS screening over getting screened with FS screening?	What do you feel are important points about FS screening that we may not have yet covered?
**Patient’s current experience of CRC screening - colonoscopy**	
What are your current feelings about Colonoscopy screening for colon cancer?	What do you know about Colonoscopy screening and what do you feel are the most important points to know about?
What do you feel are the Pros and Cons of Colonoscopy screening?	What do you feel are the important male – female differences in Colonoscopy screening? How do you feel you approach Colonoscopy screening differently than a (male, female – whichever is identified as the opposite sex)?
What do you see as the advantages of getting screened with Colonoscopy screening over not getting screened with Colonoscopy screening?	What do you feel are important points about Colonoscopy screening that we may not have yet covered?
What do you see as the advantages of not getting screened with Colonoscopy screening over getting screened with Colonoscopy screening?	
**Patient’s experience of key barriers to CRC screening adherence**	
What do you see as the major barriers for you in approaching FOBT screening?	What do you see as the major barriers for you, if you choose to obtain Flexible Sigmoidoscopy screening, in sustaining annual or biennial Flexible Sigmoidoscopy screening?
What do you see as the major barriers for you in obtaining enough knowledge about FOBT?	What do you feel are important points about major barriers in approaching Flexible Sigmoidoscopy that we may not have yet covered?
What do you see as the major barriers for you, if you choose to obtain FOBT screening, in sustaining annual or biennial FOBT screening?	What do you see as the major barriers for you in approaching Colonoscopy?
What do you feel are important points about major barriers in approaching FOBT that we may not have yet covered?	What do you see as the major barriers for you in obtaining enough knowledge about Colonoscopy?
What do you see as the major barriers for you in approaching Flexible Sigmoidoscopy?	What do you see as the major barriers for you, if you choose to obtain Colonoscopy screening, in sustaining annual or biennial Colonoscopy screening?
What do you see as the major barriers for you in obtaining enough knowledge about Flexible Sigmoidoscopy?	What do you feel are important points about major barriers in approaching Colonoscopy that we may not have yet covered?
**Patient’s experience of key factors predicting CRC screening adherence**	
What do you think and feel about the ‘effectiveness’ of FOBT? How well do you feel FOBT screening can accurately tell whether you or anyone else has colorectal cancer ?	What do you think and feel about how susceptible you feel you are to ‘getting’ colorectal cancer? In other words, how likely do you feel you are to get colorectal cancer in your lifetime? Why do you feel you are as susceptible to colorectal cancer as you have indicated you are?
What do you think and feel about the ‘effectiveness’ of Flexible Sigmoidoscopy? How well do you feel Flexible Sigmoidoscopy screening can accurately tell whether you or anyone else has colorectal cancer ?	Who do you feel influences you in terms of colorectal cancer screening? Can you talk about the influences of your family doctor, your spouse and/or members of your family? How active are these people in supporting you in obtaining colorectal screening? Do they ever oppose your getting screened? Does their support of you sometimes vary in relation to their moods or in relation to changes in your relationship with them?
What do you think and feel about the ‘effectiveness’ of Colonoscopy? How well do you feel it can accurately tell whether you or anyone else has colorectal cancer or growths called polyps that can develop into colorectal cancer?	How do you see how your personal intentions influence your attitudes and actions as far as colorectal screening is concerned? How have you thought about either FOBT or Flexible Sigmoidoscopy or colonoscopy that has influenced your decision-making or your intentions to ‘get screened’?
**Patient’s experience of gender – differences and specificities in CRC screening**	
From what you understand, do you feel there are male – female differences in approaching FOBT screening?	What do you feel are special (male or female, vary according to the gender opposite to that of the interviewee) issues in approaching Flexible Sigmoidoscopy screening?
What do you feel are special (male or female, vary according to gender of interviewee) issues in approaching FOBT screening?	What do you feel are common issues (for males and females) in approaching Flexible Sigmoidoscopy screening?
What do you feel are special (male or female, vary according to the gender opposite to that of the interviewee) issues in approaching FOBT?	Are there any additional points that we have not covered as far as gender differences are concerned, in approaching Flexible Sigmoidoscopy screening?
What do you feel are common issues (for males and females) in approaching FOBT?	From what you understand, do feel there are male – female differences in approaching Colonoscopy screening?
Are there any additional points that we have not covered as far as gender differences are concerned, in approaching FOBT?	What do you feel are special (male or female, vary according to gender of interviewee) issues in approaching Colonoscopy screening?
From what you understand, do feel there are male – female differences in approaching Flexible Sigmoidoscopy screening?	What do you feel are special (male or female, vary according to the gender opposite to that of the interviewee) issues in approaching Colonoscopy screening?
What do you feel are special (male or female, vary according to gender of interviewee) issues in approaching Flexible Sigmoidoscopy screening?	What do you feel are common issues (for males and females) in approaching Colonoscopy screening?
Are there any additional points that we have not covered as far as gender differences are concerned, in approaching Colonoscopy screening?	
**Patient’s experience of pain and anxiety reponses to CRC screening**	
How likely do you feel you are to experience pain and discomfort in association with getting an FOBT screening?	How likely are you to face true dangers in relation to Flexible Sigmoidoscopy?
How likely do you feel you are to experience anxieties in relation to an FOBT screening?	How much do you feel these experiences of pain, discomfort, anxiety and/or danger will affect your decision to get Flexible Sigmoidoscopy in the future?
How likely are you to face true dangers in relation to FOBT screening?	How likely do you feel you are to experience pain and discomfort in association with colonoscopy? Why do you feel the way you do? On what experience (your experience or the experiences of others) do you base your feelings? How likely do you feel you are to experience anxieties in relation to colonoscopy?
How much do you feel these experiences of pain, discomfort, anxiety and/or danger will affect your decision to get FOBT in the future?	How likely are you to face true dangers in relation to colonoscopy?
How likely do you feel you are to experience pain and discomfort in association with Flexible Sigmoidoscopy?	How much do you feel these experiences of pain, discomfort, anxiety and/or danger will affect your decision to get a colonoscopy in the future?
How likely do you feel you are to experience anxieties in relation to Flexible Sigmoidoscopy?	Are there any further experiences you have had or expect to have with respect to pain and anxiety in relation to any form of colorectal cancer screening that you have not talked about?
**Patient’s experience of personal decision-making in CRC screening**	
How do you go about making decisions to undergo or not undergo FOBT screening?	What about the emotions that you find you experience when you are engaged in decision-making about Flexible Sigmoidoscopy screening? Can you describe them and how they affect you?
How do you go about thinking through your decision?	If you find yourself putting off or delaying Flexible Sigmoidoscopy screening, what is that experience like for you?
What about the emotions you find you experience when you are engaged in decision-making about FOBT screening? Can you describe them and how they affect you?	How do you go about making decisions to undergo or not undergo Colonoscopy screening?
If you find yourself putting off or delaying FOBT screening, or thinking about FOBT screening, what is that experience like for you?	How do you go about thinking through your decision?
How do you go about making decisions to undergo or not undergo Flexible Sigmoidoscopy screening?	What about the emotions that you find you experience when you are engaged in decision-making about Colonoscopy screening? Can you describe them and how they affect you?
How do you go about thinking through your decision?	If you find yourself putting off or delaying Colonoscopy screening, what is that experience like for you?
**Patient’s experience of personal decision-making in other health and screening behaviours**	
How do you go about making decisions to engage or to not engage in other health enhancing or health screening activities?	How do other screening or health enhancing activities influence your approach to FOBT screening?
How do you go about thinking through your decisions to engage or to not engage in other health enhancing or health screening activities?	How do other screening or health enhancing activities influence your approach to Flexible Sigmoidoscopy screening?
What about the emotions that you find are involved when you are engaged in decision-making about other health enhancing or health screening activities? Can you describe them and how they affect you?	How do other screening or health enhancing activities influence your approach to Colonoscopy screening?
If you find yourself putting off or delaying other health enhancing or health screening activities, what is that experience like for you?	Are there any points about other screening or health enhancing activities influence your approach to FOBT or Flexible Sigmoidoscopy or Colonoscopy screening?

Participants were males and females, 50 years and above, with no prior history of CRC screening, initially contacted by random-digit-dialing who consented to study participation by phone and mail contacts. All were eligible for free-of-charge CRC screening in Ontario and each subject was assessed, using a standard interview, for CRCS screening decision stage using the following criteria: Stage 1 (never heard of the screening modality); Stage 2 (aware but not currently considering screening); Stage 3 (aware, decided against screening); Stage 4 (aware, undecided); and Stage 5 (decided to undergo screening). This staging schema, which derives from the Precaution Adoption Process Model, has been used to characterize persons eligible for CRCS according to their attitudinal proximity to action [85]. As no participants demonstrated Stage 3 (aware but decided against), the remaining stages were clustered, for reporting purposes, into unaware (Stage 1), undecided (Stage 2 and Stage 4) and decided to do (Stage 5) (see Table 2 below).

**Table 2:** Subject demographics and staging

**Females**				**Males**			
**N** = 49				**N** = 32			
**Age**: 51 – 84 years (range)				**Age**: 50 – 77 years (range)			
**Age**: 63.7 years (mean)				**Age**: 65.2 years (mean)			
**Education**:				**Education**:			
< High school: 26.5%				< High school: 29.5%			
Completed high school: 29.3%				Completed high school: 27.2%			
Completed some college/University: 23.2%				Completed some college/university: 24.3%			
Completed college/university: 21.0%				Completed college/university: 19.0%			
**Screening**^**1 **^**staging**	Females	Females	Females	**Screening staging**	Males	Males	Males
	FOBT	Flex Sig	Colonoscopy		FOBT	Flex Sig	Colonoscopy
Unaware	8.1%	8.1%	6.1%	Unaware	31.2%	34.4%	34.4%
Undecided	58.2%	44.9%	51%	Undecided	34.4%	31.2%	28.1%
Decided to do	34.7%	47%	42.9%	Decided to do	34.4%	34.4%	37.5%

**Five interview domains**							
Screening experience at time of interview	Key barriers to CRCS adherence		Key factors predicting CRCS adherence		Experience of pain & anxiety related to CRCS		Experience of gender differences in CRCS

^1^ As no participants demonstrated Stage 3 (aware but decided against), the remaining stages were clustered, for reporting purposes, into unaware (Stage 1), undecided (Stage 2 and Stage 4) and decided to do (Stage 5).

Interviews were often undertaken in two sessions, when respondents indicated needs to meet other schedule demands or reported levels of fatigue that impeded responses. The mean interview time was 1.8 hours with a range of 0.56 hours to 2.5 hours.

Care was taken to assure that participants could take the time needed to carefully consider all response options before describing experiences. All interviews were undertaken in English. Of the 81 interviews undertaken, 68 were divided into two or more sessions.

### Ethics

Approval was obtained from the Research and Ethics Board at the University Health Network in Toronto, Ontario.

### Data analysis

The demographic characteristics of the interview participants and their screening stages were tabulated (see Table 1). All interviews were audio-recorded and transcribed verbatim. The constant comparative method was used to identify key themes representing attitudes and preferences that differentiated male–female participants, with the coding of each interview’s content to identify all phrases, key words, and expressed concepts. NVivo 8 qualitative analysis software [86] aided coding the comparisons between expressed concepts identified by participants. The constant comparative method is used to denote a grounded-theory approach that analyzes interview phenomena from the ground-up, focusing on themes that emerge directly from data review, while minimizing the interpretative biases of investigators vulnerable to pre-existing theoretical orientations.

The transcripts of male and female participants were coded together, and then codes and themes were compared with respect to gender. Two unblinded team members coded all transcripts, with periodic discussions between coders and the principal investigator used to mediate discrepancies and derive final codes. Two individuals (one coder and one-non-coder) identified common themes in the data. Finally, two investigators reviewed the common themes and identified those most representative of the gender differences observed. These selections were then reviewed by all investigators and, n some cases, the descriptive terms for theme identifiers were revised.

## Results

As seen in Table 1, 49 females and 32 males of screening eligible age were interviewed about each screening modality and staged re: readiness-to-screen. A higher percentage of males were unaware of each modality at the start of interviews, although a higher percentage of females were aware but undecided. Nearly equal percentages of males and females had already decided to do FOBT, although appreciably higher proportions of females had decided to do flexible sigmoidoscopy or colonoscopy. Staging differences were minimal in males for all three modalities, while females appeared to favour both forms of endoscopy over FOBT in their readiness-to-screen.

### Dominant obstructive-cognitive themes - females

#### Bodily intrusion

Subsumed under this first theme were oral and rectal sub-themes, related primarily to colonoscopy and flexible sigmoidoscopy, and to a lesser degree, FOBT. The most notable references were to the oral ingestion of laxative mixture, perceived as directly intrusive as the actual endoscopic procedure. While the intrusion of scoping was perceived to be buffered by tranquilizing medication, laxative ingestion was seen as not buffered and as resulting in diarrhoea, gastric distress and numerous bathroom visits. In terms of scoping intrusion, most participants had difficulty imagining the level of pain relief that rendered an endoscopic procedure relatively painless. FOBT, in contrast, was seen as indirectly intrusive, given its potential unsanitariness and the messiness of managing fecal matter, resulting in exposures to pathogens no less intrusive because they originated in one’s own body.

“The purging aspect of it (ingestion of laxative mixture)… you’re the best friend of the toilet …a 24 hour process…”

(referring to colonoscopy)

“…she said it was too painful …couldn’t stand it…. it was hell … every time…I imagine she wasn’t put totally out…”

(referring to colonoscopy)

“…there’s so much discomfort and so much problems, that it should done in a hospital, not…a doctor’s office….it would be…. Intimidating…”

(referring to colonoscopy)

“…you have to drink a terrible amount of soapy stuff … so you throw it up… then you …try to get the rest… down… it’s the preparation…

(referring to colonoscopy)

“…just unsanitary….if … not done properly…you’d be contaminating things….right? …just unpleasant.”

(referring to FOBT)

“…it sounds gross. It isn’t anything I would ever choose to do….”

(referring to FOBT)

“…not something I’d like to do…I’m put off by it…”

(referring to FOBT)

#### Perforation anxiety

Separate concerns were verbalized about perforation. Women, undisturbed by the intrusiveness issues described above, expressed anxieties specific to perforation injury. Minimal distinction was made between colonoscopy and flexible sigmoidoscopy

“… somebody could…rip your colon or you could get an infection or something else … you wouldn’t run the risk of that.”

(referring to colonoscopy)

“… it sounds like it could really hurt. That’s… pretty serious…when you have … an anaesthetic….something …. to really consider.

(referring to colonoscopy)

“There have been …cases where there’s … damage to the intestinal system”

(referring to colonoscopy)

#### Embarrassment

While both males and females identified embarrassment, there was a more personalized and intense expression of embarrassment in relation to medical professionals by females.

“…I’ll just….wear a mask, or paper bag over my head so he doesn’t recognize me…”

(referring to flexible sigmoidoscopy)

“Of course, the ‘con’ would be embarrassment. I can see a lot of people …shying away …. because it’s such a personal thing. Once you have a baby though, I mean ‘hello’ (embarrassment is no longer the obstacle)”

“..I really don’t think …most doctors like doing a rectal examination. And I don’t blame them.”

“..I’ll go to the dentist and have him look down your throat…the other end… that’s very private…”

(referring to flexible sigmoidoscopy and colonoscopy)

### Dominant obstructive themes - males

#### Avoidant procrastination with underlying fatalism

While few male respondents with regular family doctors challenged their physician’s knowledge or intent in promoting screening, resistance frequently took the form of blunting and procrastinating the task of obtaining screening. This was sometimes associated with a vague but persistent notion of being fated for untreatable cancer.

“The advantage of not getting screened…. is to keep your head in the clouds.”

“What you don’t know, doesn’t hurt you….”

“I’ll call it the fear factor… do I really want to know that there’s something wrong?”

“I have this terrible thing (idea) that … it’ll kill you anyway….it’ll get you in the end… so I’d rather not [get screened] now and live my life the way I do…”

“My physician is right in suggesting it….but I keep putting it off…” (referring to colonoscopy)

“I think that men …avoid it (colorectal screening) more… because men don’t like to do …health-related things….I think men are, what’s the word I’m looking for… lazy when it comes to health issues…they believe… it’s never going to happen to them until it’s too late (referring to colorectal screening, generally)

Q. ….if you find yourself delaying health enhancing behaviours or health screening, what is the experience like?

A: “Just being lazy I guess….you know, not feeling it is that important….lazy procrastination…” (referring to colorectal screening, generally)

#### Unnecessary health care

Males, particularly those who did not maintain regular physician contact, saw CRC screening as an unnecessary component of health care. They tended to see non-symptomatic illness or illness risk as unworthy of attention. Only illnesses with disabling symptoms were seen as necessitating action. While several male respondents saw cancer and CRC, particularly, as frightening diseases, they did not sustain the idea that screening could reduce or prevent CRC impact.

Q. And what would you see as major barriers in obtaining enough knowledge (about CRC screening)?

A. “Lack of interest…if I go read everything on everything, hell, I’d be very busy. So, basically, you know, if you don’t have any kind of symptoms, I’m not going to pursue it….”

Q. What would influence you to get CRC screening?

A. Probably symptoms…I think.

(Referring to colorectal screening, generally)

“…you know unless you absolutely feel …there was something wrong and it should be checked out, I would be highly reluctant to go through it…”

(Referring to flexible sigmoidoscopy and colonoscopy)

#### Uncomfortable vulnerability

Males also indicated discomfort with the exposure of CRC screening, but more frequently mentioned the ‘position of vulnerability’ in screening, rather than being directly embarrassed in front of a specific health professional. In contrast to the personalized embarrassment of females, males seem to object to being in a vulnerable role position.

“A lot of the guys I know that have it done, they won’t say anything about it…. the females, they talk about it….there’s humility on the female side…females are smarter when it comes to health… because they don’t let their pride or sense of invasion affect them, whereas males do…it’s macho vs. humility.”

“There’s something about that area for men… it’s very vulnerable….putting yourself in that position (when being screened)…”

(Referring to colonoscopy)

“I think males would be more afraid of the body invasion than a female….because [of the] super-macho image whereas women do not have that super-macho image. They have the feeling of, if I’m sick, I want it fixed, whereas for a man, it’s ‘Hey, I'm fine.’

(Referring to colorectal screening generally)

## Discussion

The study focused on better understanding the attitudinal obstructions identified by respondents re: CRCS uptake. We specifically focused on gender divergent obstructions manifested in 4 decision-making stages, applied to 3 CRC screening modalities (FOBT, flexible sigmoidoscopy, and colonoscopy). We recruited individuals at: Stage 1 (never heard of the screening modality); Stage 2 (aware but not currently considering screening); Stage 4 (aware but undecided); and Stage 5 (decided to undergo screening).

The conceptual framework was comprehensive and its implementation required adjustments guided by three observations: 1) the great majority of respondents did not articulate as many differences as assumed observable; 2) respondents tended to adopt similar attitudes towards flexible sigmoidoscopy and colonoscopy; 3) interviewees were rarely in a divergent stage re: one modality (e.g. colonoscopy) vs. another (e.g. FOBT) (See Table 1).

The observation that participants, randomly contacted according by RDD, demonstrated attitudes less differentiated than expected, may reflect their true status, given the limits of cognitive nuance on selected topics. Some potential differentiation might have been diminished by socially desirable responding. Participants viewed the research as focused on CRC screening promotion and, at times, seemed reluctant to express responses indicating negative attitudes and rejections of CRC screening.

The gender differences observed were neither closely tied to screening stage nor modality. Women, as predictable from previous findings, reported more consistent physician relationships and appeared more knowledgeable about CRC screening, and better able to articulate views on screening and screening decisions [21,24,87-90]. Men reported less consistent physician relationships, were less knowledgeable and often kept decision-making processes vague and emotionally distanced (i.e. at ‘arm’s length’).

Male-health researchers have speculated that masculine-specific values, constructed during male-to-male social interactions, significantly reduce male health [23,28,29,91-98]. Some hypothesize traditional masculine roles as ‘health hazards’ [94] based on observations that redundant tendencies towards stoicism and emotional suppression, transmitted during masculine-role socialization, are particularly influential in illness and health-related behaviours[93]. Adherence to other masculine role characteristics, like excessive self-reliance and dominance, further impede appropriate health service accessing [21,87,88,92,94,98,99].

The specific kind of vague processing observed in male participants aligns with the blunting construct where anxiety-provoking situations are distanced emotionally and processed ambiguously, with fragmentation of content and timing [100-103]. Rather than acknowledge anxiety and the need to decide, men blunt by distancing anxiety-provoking issues, denying their possible importance and ambiguously referring to timelines for effective action. The blunting construct overlaps with the construct of alexithymia, which defines a more general deficiency in understanding, processing, and describing emotions [104-107]. Males have a higher prevalence of alexithymia (than females) and, when combined with typical socialization patterns, alexythymic-factors can contribute to a greater tendency towards poor screening health practices [104,107,108].

According to both blunting and alexythymic perspectives, when emotions related to decision-making remain ambiguous, actions on one’s behalf (involving confrontation with risk, distress and anxiety) are impeded [107,109,110]. Avoidant procrastination appeared to be a fundamental male characteristic with respect to CRCS. In literature on male socialization, adherence to a ‘stoic’ role impedes disease prevention, as anxiety (about potential disease) is often a motivational factor in protective actions [111,112]. While few males expressed a rejection of CRC screening, males, generally, were less expressively favourable to screening than females. When they demonstrated favourable attitudes, they often saw themselves as simply adhering to physician directions.

Past female-specific CRCS research has indicated aversion (when compared to men) to endoscopic screening (internal scoping in flexible sigmoidoscopy or colonoscopy) and FOBT (fecal sampling without internal scoping) [35-45]. Comparatively speaking, women were observed to more frequently arrive at definite screening decisions, based on protecting personal health, or a delay because procedures were perceived too distressing. Men, in contrast, were reluctant to express outright screening rejections, instead vaguely describing themselves as ‘in the process of’ decision-making and action.

The impeding female attitudes were related to high levels of distress over the physically intrusive, anxiety provoking aspects of CRCS. Anxiety-sensitivity is an applicable construct within current psychological literature, describing a state where problems are avoided because of fears of anxiety-related sensations, based on cognitions about harmful anxiety effects [113-120]. When impeded by anxiety-sensitivity, people hyper-react to immediate stress, feeling stressors do more harm than the good derived from stress-mastery. When gender differences have been tested regarding anxiety-sensitivity, females demonstrate a higher prevalence, with respect to the global construct and tend to score high specifically, on the physical concerns subscale that measures potential physical harm related to anxiety experiences [121]. This describes the state of female participants who rejected screening, not solely because of intrusiveness, perforation fears or embarrassment, but because the associated anxiety-related distress seemed too powerful to even attempt mastery. While lower level anxieties (i.e. worry) may be conducive to screening adherence, as indicated in a recent study on the facilitating effects of worry in breast screening, levels perceived by females as extreme appear to impede screening [122].

Women who have not had CRCS-related endoscopy frequently state as reasons the fear of pain, the fear of positive results, embarrassment, inconvenience, expense and the absence of physician recommendation [8,19,42,123,124]. References to pain are better understood in light of an established finding that females regularly report more pain/discomfort with endoscopy (than men) linked to anatomical differences, although this finding does not account for sedation effects which may attenuate such differences [40]. Additional factors that appear contributive to female endoscopy rejection revolve around embarrassment. Many females prefer female endoscopists [41,43-45,125] and some investigators suggest the availability of more female endoscopists would yield higher screening rates, supported by evidence that women who prefer female endoscopists are less likely to screen [125]. This preference could be addressed by medical schools and residency programs offering fellowships, salary incentives, or other recruitment motivators for female physicians to receive specialty training in gastroenterology.

In terms of CRCS awareness, women have the advantage of more exposure to cancer screening than men, given the recommendations of annual/bi-annual cervical screening (Pap testing) and breast screening (annual/biannual mammography) [126,127]. Existing data supported the facilitative character of these cancer screening modalities as several studies indicate women who participate in breast cancer screening are more likely to participate in CRCS [43,126,128-130].

In applying these findings to screening promotion, the data suggest males should be supported in taking definitive ‘stands’ on screening, given tendencies to ambiguously procrastinate. Tailored promotion may already perform this function (by engaging individuals in systematic decision-making). But the injunction to ‘screen now, not later’ can additionally counteract tendencies to delay and render ambiguous screening timelines. On the other hand, from responses observed, males seem to require a concerted effort aimed at alerting them to the need for disease prevention practices altogether. They seem mired in thinking that only symptomatic and disabling medical problems require care. If this finding is accurate, a deliberate effort to change these fundamental attitudes in support of preventive care is required. For females, their readily expressed anxieties and anxiety-sensitivity could be counteracted by injunctions to focus on the relatively limited interval of distress involved (‘just a few hours could save your life’). With the higher proportions of breast screening adherence, reference could be made to similarly overcoming CRCS anxieties (‘if your breasts are screened, why not your colon?). Other themes worth emphasizing in counteracting female obstructive cognitions are the very low risks associated with colonoscopy, especially when compared to the sizeable associated reductions in CRC risks. This theme can be amplified by referring to the extensive training of colonoscopists and the fact that variably sized colonoscopes can overcome the differences associated with female anatomical factors. In terms of FOBT, emphasis on the specific design features of kits that emphasize cleanliness and prevention of contamination are important. Once again, statistics verifying that these features effectively reduce risks of infection would be further reassuring.

Fatalism, in our data, appears an obstructing attitudinal factor in CRCS in males, confirming existing data suggesting fatalism is an important negative factor in cancer screening in males and females. Current literature suggests elderly individuals, members of ethnic minorities, and individuals from lower socioeconomic backgrounds are particularly susceptible to fatalistic perspectives that prevent or retard cancer-prevention. As our data accented fatalism in males, it’s notable that other studies have also detected a relatively high prevalence of fatalism in males that impedes health preventive and precautionary behaviours [131]. The precision of measurement of fatalistic attitudes has been aided by the development and psychometric validation of instruments like the Powe Fatalism Inventory [132] and further study with more precise investigation is warranted. From our perspective, fatalism could be a factor that influences men to procrastinate and render ambiguous screening schedules and activities. On the other hand, according to the theory of cognitive dissonance, the behavioural delays of screening might reciprocally contribute to the attitude that screening is ineffective, in rationalizing the lack of attentiveness and precaution manifested behaviourally.

In terms of study limitations, the original conceptual framework motivated a plan apportioning 15 participants per gender and screening stage for a total of 120 interviewees (60 males and 60 females). We were only able to recruit and consent about two-thirds of the sample target (N = 81) and were especially hampered by the difficulty of recruiting males. In terms of the obtained sample, we acknowledge that a mean age of 63.7 years (for females) and 65.2 years (for males) may appear to indicate too much emphasis on the more senior decades of CRC screening-eligibility, considering the need for attention devoted to people in their first decade of screening eligibility. The skew in our data was related to being specifically required to not enact an upper limit age by the Canadian Institute for Health Research and can be considered a study limitation. This sampling limitation, however, relates to the concept of saturation in qualitative research which is defined by the threshold reached in data collection when no new information is identified on a specific topic, within a defined subject group. When saturation is reached, additional interviews only yield redundant data rather than novel findings. One controversy regarding saturation involves how many additional participants should be interviewed to confirm no additional information is forthcoming. For example, after concluding 5 interviews that indicate saturation has been reached, a 6th interview might yield novel data. While the subsamples of interviewees (32 males and 49 females) seemed sufficient to achieve saturation, and saturation was observed in the overall gender-specific samples, the participants recruited per screening stage and gender were typically less than N = 15 (the number of interviews often associated with saturation). It is possible then that had we recruited greater numbers of participants, saturation would have been further confirmed, in terms of gender per screening stage. This level of precision awaits future studies that likely will confront the difficulties we encountered. The response rate of contacted individuals through RDD was low (< 2%) meaning that 100 calls yield 2 eligible, consented participants and a total greater than 4000 calls were necessary.

## Conclusion

Marked differences were observed in obstructive CRCS cognitions per gender. While females articulated reservations about CRCS-associated distress, males suppressed negative views but ambiguously related to the task of completing screening. Their procrastination sometimes masked a deeper fatalism about cancer disease and disbelief in the preventive-protective elements of screening. While both genders cited embarrassment as an obstacle, female perceptions were personalized (identifying a health provider) while males more generally referred to vulnerability without envisioning a specific person encountered.

## Competing interests

The authors declare that they have no competing interests.

## Authors’ contributions

PR, REM, LP, DP and LR drafted the manuscript. PR and DP conducted analyses. PR, REM, LP and LR drafted semi-structured interview questions. All authors read and approved the final manuscript.

## Pre-publication history

The pre-publication history for this paper can be accessed here:

http://www.biomedcentral.com/1471-2458/13/500/prepub
